# mTOR Signaling at the Crossroad between Metazoan Regeneration and Human Diseases

**DOI:** 10.3390/ijms21082718

**Published:** 2020-04-14

**Authors:** Yasmine Lund-Ricard, Patrick Cormier, Julia Morales, Agnès Boutet

**Affiliations:** Centre National de la Recherche Scientifique (CNRS), Sorbonne Université, Integrative Biology of Marine Models (LBI2M), UMR 8227, Station Biologique de Roscoff (SBR), 29680 Roscoff, France; ylundricard@sb-roscoff.fr (Y.L.-R.); patrick.cormier@sb-roscoff.fr (P.C.); julia.morales@sb-roscoff.fr (J.M.)

**Keywords:** mTOR pathway, regeneration, whole-body, appendage, muscle, axon, epidermis, liver, kidney, autophagy, proliferation, stem cell, differentiation, human diseases

## Abstract

A major challenge in medical research resides in controlling the molecular processes of tissue regeneration, as organ and structure damage are central to several human diseases. A survey of the literature reveals that mTOR (mechanistic/mammalian target of rapamycin) is involved in a wide range of regeneration mechanisms in the animal kingdom. More particularly, cellular processes such as growth, proliferation, and differentiation are controlled by mTOR. In addition, autophagy, stem cell maintenance or the newly described intermediate quiescence state, G_alert_, imply upstream monitoring by the mTOR pathway. In this review, we report the role of mTOR signaling in reparative regenerations in different tissues and body parts (e.g., axon, skeletal muscle, liver, epithelia, appendages, kidney, and whole-body), and highlight how the mTOR kinase can be viewed as a therapeutic target to boost organ repair. Studies in this area have focused on modulating the mTOR pathway in various animal models to elucidate its contribution to regeneration. The diversity of metazoan species used to identify the implication of this pathway might then serve applied medicine (in better understanding what is required for efficient treatments in human diseases) but also evolutionary biology. Indeed, species-specific differences in mTOR modulation can contain the keys to appreciate why certain regeneration processes have been lost or conserved in the animal kingdom.

## 1. Introduction

**Figure 1:**
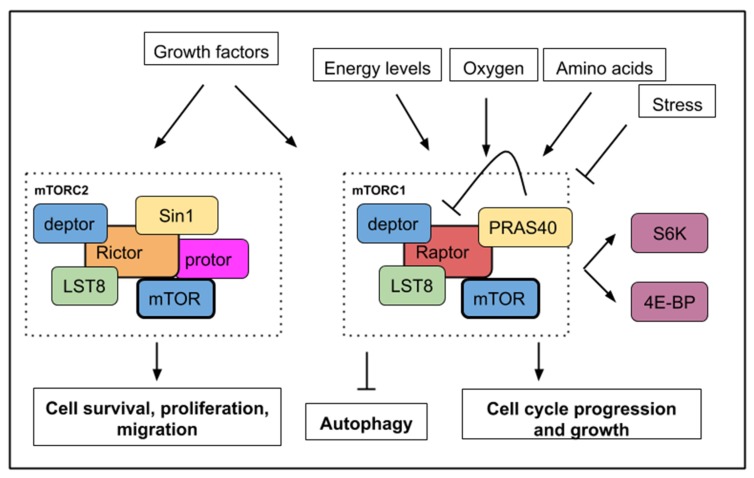
A simplified mTOR (mechanistic/mammalian target of rapamycin) pathway with upstream signals, which activate or inhibit mTORC1 or mTORC2 activities. mTORC1 activity is sensitive to growth factors, energy levels, oxygen, amino acids, and stress while mTORC2 activity responds to growth factors only. Below, the main cellular processes, which are affected by mTOR activity. mTORC1 activity leads to cell growth, cell cycle progression with an increased phosphorylation of S6K1/2 (S6 kinase 1/2) and 4E-BP (4E binding protein). mTORC1 activity inhibits autophagy. mTORC2 activity controls cell survival, proliferation, and migration.

The mTOR signaling pathway is highly conserved in eukaryotic organisms including unicellular photosynthetic eukaryotes [1], while the iconic developmental pathways (e.g., FGF, Hedgehog, Wnt/β-catenin, TGF-β, Notch pathways) operating in tissue modeling both at the embryonic and adult stage are only observed in metazoans. With the components of these developmental pathways assembled early in metazoans [2], the mTOR signaling pathway might have played a more ancestral role in the emergence of the eukaryotic cell and its metabolism. Protein and nucleotide synthesis are important anabolic processes targeted by mTOR signaling together with cell growth, proliferation, cell survival, and migration. The catabolic process of autophagy (Table 1) is another cellular function regulated by mTOR activity. Nutrients, energy, oxygen, growth factors, glucose, cytokines rank among signals within the cell microenvironment that are sensed and integrated by the mTOR pathway to produce a suitable response (Figure 1). TOR is the catalytic subunit of two distinct protein complexes TORC1 and TORC2 containing Raptor (regulatory-associated protein of mammalian target of rapamycin) and Rictor (rapamycin-insensitive companion of mTOR) respectively. Rapamycin (or sirolimus) is a potent inhibitory molecule that targets mTORC1. This small molecule was first identified from a soil bacterium *Streptomyces hygroscopicus* in 1972 from Rapa Nui (Easter Island). mTORC2 is insensitive to acute rapamycin treatment but chronic exposure can disrupt its structure. Raptor and Rictor protein scaffolds participate in assembling the different constituents of the complexes and binding substrates.

Detailed data about the structure, components and regulation of both TORC1 and TORC2 complexes are available in several recent reviews [3,4,5]. S6K1/2 (S6 kinase 1/2) and 4E-BP (4E binding protein) constitute important substrates phosphorylated by the serine/threonine kinase TOR to initiate protein translation and are commonly used as readout for TORC activity. LST8 (lethal with SEC13 protein 8) and deptor are present in both complexes. PRAS40 (Proline-rich Akt substrate 40 kD) is a specific inhibitor of mTORC1 [6] while Sin1 (stress-activated map kinase-interacting protein 1) is a regulatory component of mTORC2 [7] (Figure 1).

Regeneration ability is an endogenous tissue or organ-specific characteristic. In metazoans, a large diversity of mechanisms is involved to repair or regrow the missing structure, organ or body part [8]: For example, the use of resident stem cells and committed progenitors, the transdifferentiation of existing cells that are reprogrammed and finally the cell cycle reactivation of resident differentiated cells (Table 1). During organogenesis, the mTOR pathway is paramount in regulating physiological cell growth and proliferation [3]. In adult animal organisms, mTOR signaling is involved in diverse processes of tissue/body regeneration. Studies that provide a mechanistic understanding of the regeneration processes contribute to the strategies that can stimulate regeneration at sites where regenerative failure is the norm. Regenerative failure has been shown to be linked to the microenvironment of the healing wound and not to an inability of cells to respond in a pro-regenerative manner [9,10,11,12,13,14,15]. This review will focus on the role of the mTOR pathway in regeneration. With selected examples from different tissue types, data will be presented on the ways in which mTOR controls cellular behaviors to contribute to regeneration. This will highlight how and where this pathway could be modulated to improve the restoration of disease-induced damages.

## 2. mTOR Signaling Involvement during Appendage Regeneration

Many vertebrate species are capable of impressive feats of regeneration such as limb, tail or fin regeneration. Adult salamanders and teleosts have a higher regenerative potential as they are able to regenerate whole limbs and fins, which host a series of specialized tissues such as epidermis, nerves, bone, endothelium, soft mesenchymal cells, and fibroblast-like cells. For limbs, amputation is followed by the migration of epidermal cells to cover the wound and a blastema is formed, reminiscent of the embryonic limb bud. Caudal fin regeneration in zebrafish also implies wound covering by skin cells and blastema setup. Within this blastema, mesenchymal cells and osteoblasts proliferate a few hours after the onset of amputation. In mammals, regeneration in which the structure of lost tissue is recapitulated appears to be limited to the distal tip of the digit [9]. In mouse, the regeneration process has shown the conservation of the same steps of wound closure, blastema setup, cell proliferation and differentiation [9].

A study using the suppression subtraction hybridization (SSH) technique combined with bioinformatics analyses was performed on a freshwater teleost following fin amputation. Genes involved in TOR signaling were identified as differentially expressed during the caudal fin regenerative process [16]. It, therefore, appears that the mTOR pathway is important in controlling the process of appendage regeneration, which involves the proliferation and remodeling of numerous cell types.

**Figure 2:**
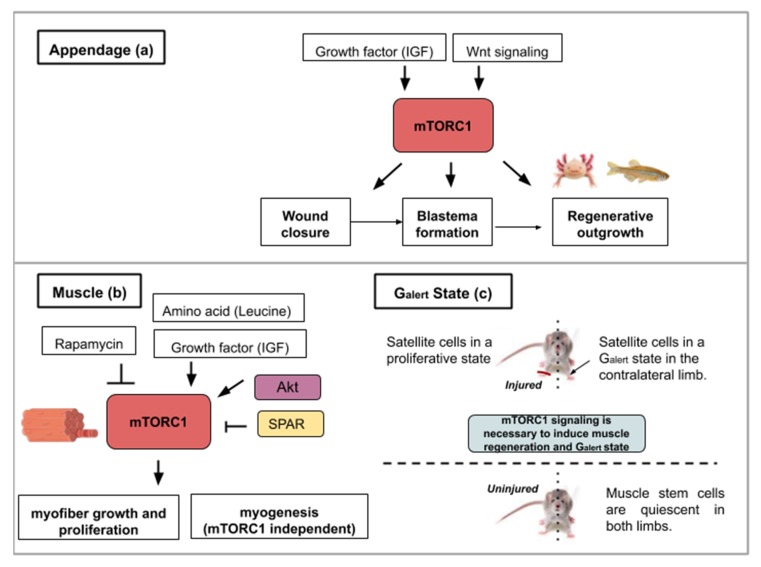
This figure illustrates the ways in which activity from the mTOR signaling pathway contributes to appendage (**a**) or muscle regeneration (**b**,**c**): (**a**) Concerning appendage regeneration, the Wnt/β-catenin pathway and insulin-like growth factor (IGF-1) activate mTORC1. This activity leads to the wound covering, blastema formation and regenerative outgrowth of the appendage. (**b**) During muscle regeneration, mTORC1 activity is necessary for myofiber growth but not myogenesis. mTORC1 inhibition by rapamycin treatment inhibits regeneration whereas the leucine amino acid, insulin-like growth factor 1 or Akt activity contribute to mTORC1-mediated muscle regeneration. SPAR regulatory protein (small regulatory polypeptide of amino acid response) can inhibit mTORC1 activity and hinder muscle regeneration. (**c**) mTORC1 signaling is necessary for muscle regeneration in the injured limb and to induce a G_alert_ state in the contralateral limb. The G_alert_ cells enter the cell cycle more rapidly and show an increase in size compared to quiescent satellite cells.

Following caudal fin amputation in zebrafish, TOR signaling, reflected by the levels of the phosphorylated form of S6K (S6 Kinase), is active in wound epidermal cells, osteoblasts and proliferative proximal region of the blastema [17] (Figure 2a). During regenerative outgrowth, mTORC1 inhibition (with rapamycin treatment) suppresses blastema formation. mTORC1 is required for cell proliferation involved in blastema formation and then for cell survival. Additionally, the mTORC1 function was shown as an osteoblast proliferation and differentiation factor. Using the same model, pharmacological inhibitions of Wnt/β catenin and IGF (Insulin-like Growth Factor) pathways block cell proliferation and regeneration indicating that TOR activation is under their control [17] (Figure 2a). These results show that growth factors like IGF acts as an mTOR activity-promoting signal and that other pathways like the Wnt/β-catenin pathway, can also interact with the mTOR pathway and alter the regenerative response.

In the regeneration examples detailed above, growth factors are efficient mTOR activity-promoting agents. mTOR activity is necessary for cell proliferation, survival, differentiation, and growth during appendage regeneration. Molecular crosstalk such as exists between the Wnt/β catenin and mTOR pathways is an important element to consider in designing drugs that enhance regeneration. These examples of appendage regeneration are on the more spectacular end of the regenerative spectrum of the animal kingdom. Studies with tissue-specific regeneration in animal models after injury have an easier framework for which to extract data useful for treating human diseases. mTOR roles in specific cell types will be investigated separately in the next paragraphs along with how mTOR modulations can alter the microenvironment in a pro-regenerative fashion.

## 3. Adult Myofiber Growth and Formation Is Mediated by mTOR Signaling

In muscle tissue, damages and atrophies are caused by mechanical shock or genetic diseases and can seriously disrupt muscle functions. Chronic deterioration of muscular tissue is also observed during the aging process. This makes the development of efficient therapies for muscle regeneration of particular interest. Muscle regeneration is an event that is composed of two main steps: myofiber neoformation and fiber growth [18]. In chordates, adult skeletal muscles possess their own stem cell population, the myogenic satellite cells. These cells are positive for the myogenic Pax7 transcription factor and are involved in muscle repair, growth, and regeneration. The existence of Pax3/7-expressing muscle satellite cells has also been discovered in the crustacean *Parhyale hawaiensis* suggesting that this stem population was present in the bilaterian common ancestor [19]. With the cellular basis for muscle regeneration being evolutionarily conserved between arthropods and mammals, research benefits from the use of animal models with regenerative capacities in deciphering the way in which the mTOR pathway can serve or disserve regeneration. Examples of mTOR involvement in regeneration will be illustrated below with mouse and axolotl (amphibian) models.

mTOR activity is involved in homeostatic myogenesis and is associated with enhanced muscle regeneration. The role of TOR signaling has been genetically demonstrated using a mouse model harboring a conditional deletion of *TOR* in satellite cells [20]. Upon skeletal muscle injury, these mice display necrotic fibers and fail to activate proliferation in satellite cells (Figure 2b). The myogenic program is also affected by TOR deletion as shown by the reduced expression of *Myf5*, *MyoD* and *MyoG* gene products in myoblasts [20]. Using transgenic mice in which Akt is constitutively active, Lai et al., investigated changes in muscle mass [21]. Akt (also known as PKB) is a serine/threonine-specific protein kinase that activates mTORC1. Akt participates in several processes such as glucose metabolism, apoptosis, cell proliferation, transcription, and cell migration. Constitutive activation of Akt and by extension mTORC1 in transgenic mice results in skeletal muscle hypertrophy [21].

In contrast, in wild type adult mice, the addition of rapamycin inhibits muscle regeneration after myotoxin exposure. This result shows mTORC1’s involvement in muscle regeneration. Investigating the properties of adult pig satellite cells, Han et al. found that muscle growth (protein synthesis and proliferation) in vitro is highly dependent on mTOR signaling activation after leucine and insulin-like growth factor 1 (IGF-1) stimulation [22] (Figure 2b). Supplementation of amino acids like leucine [23] or delivery of factors containing insulin-like growth factor 1 [24] have been successfully tested on rats and mice as a means to ameliorate muscle regeneration. These studies show the necessity of mTOR activity in adult satellite cells for proper stem cell activation and myofiber growth, which are essential in muscle development and regeneration.

Complementarily to myofiber growth, myofiber formation is an important process in muscle regeneration. To dissect out the kinase activity of TOR in skeletal muscle repair, transgenic mice with an inactive TOR kinase in skeletal muscles were designed [18]. This study revealed that myofiber growth was impaired but not the formation of nascent myofibers. These data suggest that TOR-regulated muscle regeneration displays kinase-dependent and -independent mechanisms [18] (see Figure 2b).

More recently, a long non-coding RNA (lncRNA), highly expressed in mouse skeletal muscles, was found to have the property to modulate TORC1 complex activity [25]. This lncRNA, called LINC00961, is conserved in mice and humans [25] and has the particularity to host a small open reading frame that is translated into a protein (SPAR, small regulatory polypeptide of amino acid response) displaying co-localization with late endosome and lysosome markers in both HeLa and prostate cancer cell lines. The TORC1 complex is a nutrient sensor, able to move from the cytoplasm to the lysosome after amino acid stimulation. Investigating the effect of SPAR on amino acid-induced TORC1 activation, SPAR was discovered to impair mTORC1 correct localization to the lysosomes [25]. Spar-deficient mice (Spar^−/−^) display a better record in tibialis anterior muscle regeneration compared to wild type mice (larger and more mature myofibers) and showed increased phosphorylation of S6K1/2 and S6 (see Figure 2b) [25]. This reflects a hyperactivation of mTORC1 complex when SPAR is knocked out in skeletal muscles. SPAR functions to negatively regulate mTORC1 recruitment, through interaction with v-ATPase. The modulation of regulatory elements of mTOR activity represents another possible method to enhance endogenous regeneration. A way to therapeutically boost myofiber proliferation and growth during muscle repair could then be to negatively target this atypical polypeptide hidden in long non-coding RNA.

Finally, in a study analyzing mouse satellite cells located in the contralateral muscle after injury with myotoxins, mTORC1 was discovered to control a novel state within the G_0_ quiescence phase newly termed G_alert_ [26]. Two cell populations were compared; the muscle stem cells from the site of injury and those of the contralateral limb. This study found that these cells enter the cell cycle more rapidly and display an increase in cell size compared to quiescent satellite cells. These two parameters (cell size and cell cycle entry speed) are not as elevated as in satellite cells from the injured muscle but in a kind of intermediate state (G_alert_) (see Figure 2c). mTORC1 signaling is necessary and sufficient to induce this “alert” response in satellite cells. The authors proposed that the quiescence status of stem cells actually comprises several phases. This G_alert_ state might allow stem cells to respond more rapidly in case of injury and would then constitute a form of cellular memory as the one observed in cell-mediated immune response [26].

Interestingly, similar contralateral activation of mTOR was observed in the axolotl limb amputation model [27]. The phosphorylation of S6 ribosomal protein, one of the main substrate of S6K1, is slightly but significantly increased in the satellite cells of the contralateral intact limb compared to levels observed in the limb of non-amputated animal [27]. The precise upstream signals, which activate mTORC1 and enable cell-state transition, remain to be deciphered. Nonetheless, these remarkable findings on an apparent systemic cellular activation response that occurs distant to the site of injury could guide future research in regenerative medicine.

Altogether, these data demonstrate that TORC1 activity is necessary to achieve the correct process of muscle regeneration for cell growth and cell proliferation. The regeneration-competency of satellite cells is also a factor that will influence the degree of recovery. The previously described G_alert_ cell state, which is under the control of mTOR activity, serves as an illustration for improved competency. Any diseases displaying chronic muscle degradation might, therefore, consider inducible activators of TORC1 as suitable therapeutic molecules such as growth factors, amino acids or modulators of the regulatory elements of mTOR.

## 4. The mTOR Signaling Roles during Axon Regeneration

### 4.1. The mTOR Signaling Roles in Vertebrate Retinal Neuron Regrowth after Axotomy

In general, axons after injury do not spontaneously regenerate in the adult mammalian central nervous system. Interestingly, the mTOR pathway is involved in the differentiation of human retinal ganglion cells from induced pluripotent stem cells (iPS cells) [26]. This model of neurogenesis has made evident that function and neuritogenesis of human retinal ganglion cells (RGCs) are dependent on mTOR [28,29]. In addition, high levels of the phosphorylated form of S6 (pS6) are observed in the embryonic chick retina as progenitors cells differentiate [30]. These studies show that mTOR signaling participates in the in vitro proliferation and differentiation of retinal stem cells. Upon retinal injury in adult chicks, Müller glia cells (a type of retinal glial cell) can proliferate and give rise to a population of progenitors (Müller glia-derived progenitor cells, MGPCs) that can be converted into retinal neurons. This valuable property of Müller cells is observed in teleosts but strongly reduced in amniotes. In adult chicks, retinal damage induced by NMDA (*N*-methyl-d-aspartate) is followed by the activation of TOR signaling in Müller glia and this activation is necessary for Müller glia-derived progenitor cells’ proliferation [30]. This suggests that regeneration mechanisms observed in adult nerve cells are reminiscent of the machinery running during development. Targeting mTOR signaling in adult cells could thus potentially stimulate this developmental reactivation.

**Figure 3:**
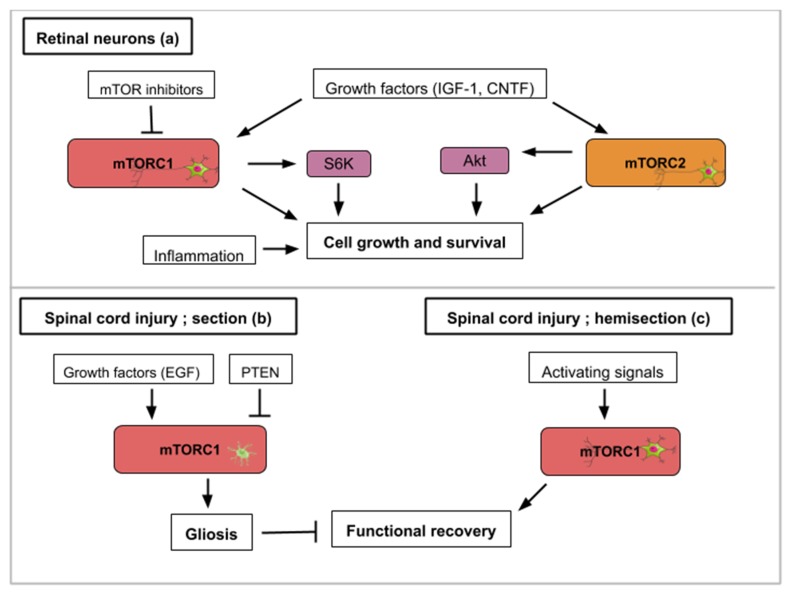
This figure illustrates how activity from the mTOR signaling pathway contributes to axon regeneration in the selected examples of retinal nerve damage (**a**) and spinal cord injury (**b**,**c**): (**a**) Concerning retinal nerve regeneration, mTOR activity enhances axon elongation and survival leading to functional regeneration. Signals that upregulate mTOR activity like PTEN inhibition, siRTP801, Pam_3_Cys (pro-inflammatory signal), CNTF (ciliary neurotrophic factor) or IGF-1 lead to axon recovery. TOR-independent axon recovery exists however as rapamycin-treated PTEN-deleted neurons and Pam_3_Cys untreated neurons still showed recovery. (**b**) For spinal cord injuries, mTORC1 activity in astrocytes hinders neuronal recovery with the formation of glial scars (gliosis). Growth factors like EGF (epidermal growth factor) can activate mTORC1. (**c**) On the contrary, mTORC1 signaling in hemisection spinal cord injuries promotes growth and functional regeneration.

PTEN and TSC1/2 are two important molecular actors regulating the mTOR pathway. PTEN is a phosphate and tensin homolog, which acts upstream of mTOR as an inhibitor. TSC1/TSC2 or Tuberous sclerosis proteins 1 and 2 form a protein complex that inhibits mTOR activity. Retinal ganglion cells (RGCs) in the adult mouse normally degenerate after section of the optic nerve. Hypothesizing that manipulation of the intrinsic properties of retinal neurons can change their ability to regrow after axotomy, mice carrying the deletion of the lipid phosphatase PTEN in adult retinal ganglion cells were generated [31]. These mice were able to regrow their optic axon indicating that TOR activation might be a crucial step in this process. According to molecular crosstalk, the mTOR pathway is englobed by a larger PI3K/AKT/mTOR pathway. Thus, as a consequence of *PTEN* deletion, Akt is strongly activated and the kinase GSK3β appears to be a core component of the Akt-induced axon regeneration [32] (Figure 3a). Similarly, another study used small interfering RNA (siRNA) to invalidate RTP801 (another inhibitor of TOR induced upon hypoxia and stress conditions) (Figure 3a). RTP801 (also known as Redd1) is a stress-induced protein that suppresses mTOR signaling by stabilizing TSC1/TSC2 (inhibitors of mTORC1/2). Intravitreal injection of this mTOR activating siRNA was found to promote the release of several neurotrophins and RGC neuroprotection [33]. A study combining PTEN inhibition (PTEN KO mice) with ambroxol treatment demonstrated significant enhancement of axonal outgrowth in dorsal root ganglion (DRG) neurons [34]. Ambroxol was shown to increase the expression of a transcriptional network that coordinates central nervous system regeneration [34]. Altogether, these data show that a constitutive activation of the mTOR pathway promotes both retinal ganglion cell survival and axon regrowth.

In adult mice, inflammatory stimuli that activate resident retinal glial cells (astrocytes/Müller cells) greatly favor axon regeneration of retinal ganglion cells [35]. Indeed intravitreal application of Pam_3_Cys, an agonist of toll-like receptor that constitutes an inflammatory stimulus, impairs the decrease of TOR activity normally observed after optic nerve crush in wild type mouse (Figure 3a). Surprisingly the inhibition of TOR (rapamycin treatment) does not modify the conversion of optic neurons from a non-regenerative to a regenerative status upon inflammatory stimulation meaning that additional signaling pathways can take over this intrinsic neuron property. Thus, mTOR activity is not generally required for neuroprotection or switching mature neurons into an active regenerative state, but it is important for the maintenance of the axonal growth state [35].

Studies that focus on the mechanistic explanations behind optic nerve axon regeneration have revealed which mTOR components are most likely involved. In the mouse optic nerve crush model, mTORC1 downstream effectors (S6K1 and 4E-BP) are differentially involved. S6K1 activation after crush clearly induced axon elongation and survival of optic neurons. In contrast, the deletion of 4E-BP (4E-BP1/2 double KO mice), which is normally phosphorylated and inhibited upon TOR activity, had no effect on regrowth [36] suggesting that S6K1 or other TOR substrates are operating. It is also worth mentioning that axon recovery upon optic nerve damage is still observed on *PTEN* deleted neurons when they are treated with rapamycin suggesting the implication of mTORC1-independent pathways [31]. Since PTEN deletion constitutively activates mTOR signaling but rapamycin inhibits mTORC1 activity, this suggests an mTORC2-mediated recovery (Figure 3a). mTORC2 controls Akt, which regulates cellular processes such as metabolism, survival, apoptosis, growth, and proliferation. Rapamycin-resistant axon recovery and TOR-independent mechanisms of axogenesis are also observed in peripheral sensory neurons [23,24].

Finally, when considering retinal neuron axon regeneration, outgrowth following injury cannot be the only investigated parameter in a human disease-context. Juvenile and adult mice with either PTEN and SOCS3 (suppressor of cytokine signaling 3) co-deletion, or co-overexpression of osteopontin (OPN)/insulin-like growth factor 1 (IGF1)/ciliary neurotrophic factor (CNTF), showed regrowth of retinal axons and formation of functional synapses in the superior colliculus (SC), but not significant recovery of visual function [37]. PTEN and SOCS3 deletions as well as the growth factors’ (OPN, IGF1 and CNTF) overexpression activate mTOR signaling (Figure 3a). The regenerated axons failed to conduct axon potentials from the eye to the brain. With functional recovery in mind, the administration of voltage-gated potassium channel blockers was shown to restore conduction and results in increased visual acuity [37]. When rebuilding circuits and evaluating regeneration after optic nerve injury, both the axon regrowth and proper myelination seem to be required. In general, for each regeneration challenge, attention must be paid in the specificities required for functional regeneration.

The medical challenge resides in finding the exact ways to stimulate mTOR activity to maximize the optic nerve’s regenerative properties. As exposed in this review, current leads include inhibitors of mTOR inhibitors (PTEN, RTP801 or TSC1/TSC2), mTOR activators and growth factors. Conditions like glaucoma where the optic nerve is damaged and which is the leading cause of blindness in people over 60 could benefit from such treatments.

### 4.2. The mTOR Pathway Mediates Drosophila Neuron Regrowth and Remodeling during Metamorphosis

The TOR pathway is also involved in axon regrowth in *Drosophila*. During metamorphosis, neurons from the mushroom body are reshaped. This consists in parceling exiting axons followed by the re-extension of new ones from the cellular body. This phenomenon mimics cellular events occurring after axotomy in neuron and is different from initial axon growth from a newborn neuron. During mushroom body remodeling, axon regrowth is mediated via the TSC-Rheb-TOR pathway, which is activated by the nuclear receptor UNF [38]. In this system, TOR is also regulating neurite sprouting but its activation is under the control of PI3K/PTEN [39].

### 4.3. The Cell-Specific mTOR Signaling Roles in Mammalian Spinal Cord Neurons

Spinal cord injuries (SCI) have been connected to mTOR signaling in different ways. The spinal cord connects the brain to the peripheral nervous system. The mTOR pathway has been shown to promote or inhibit axon regeneration in SCI animal models. Most frequently, two types of SCI animal models are used; spinal cord injury and spinal cord hemisection with the former aiming for complete anatomical and functional damage of the spinal cord while the latter is an incomplete injury with a certain remaining nerve connectivity. Glial scars, formed by the activation of inactive astrocytes, involve the proliferation or hypertrophy of astrocytes, which create a physical and chemical barrier that hinders neuronal recovery [40]. The conversion of astrocytes depends on epidermal growth factors (EGF), which activates the Rheb/mTOR pathway [5]. Overexpressed PTEN attenuates gliosis at three days after SCI and enhances motor functional recovery [5] (Figure 3b). In general, in astrocytes, a hyperactive mTOR is a negative regulator for recovery following SCI [41]. Restravol is an example of an mTOR inhibiting drug that has been successful in restoring nerve function following SCI [42]. However, in spinal cord hemisection, mTOR activation efficiently promotes regrowth and regeneration of the corticospinal tract [5] (Figure 3c). In dorsal root ganglion neurons, mTOR activation increases regrowth and recovery following peripheral nervous system injury [43]. Diseases like amyotrophic lateral sclerosis (ALS), which specifically affects motoneurons, could gain from treatments which alter mTOR activity in the spinal cord. To recapitulate, hyperactivity of mTOR in astrocytes is correlated to an inflammatory response that is deleterious for neuron recovery while mTOR activation in spinal cord neurons enhances axon regeneration. Importantly, functions of mTOR in varying neuronal injury models can be different or even opposing, which might be attributed to the type of cell in which the mTOR activity takes place.

## 5. The mTOR Signaling Roles in Wound Healing during Epidermis Regeneration

**Figure 4:**
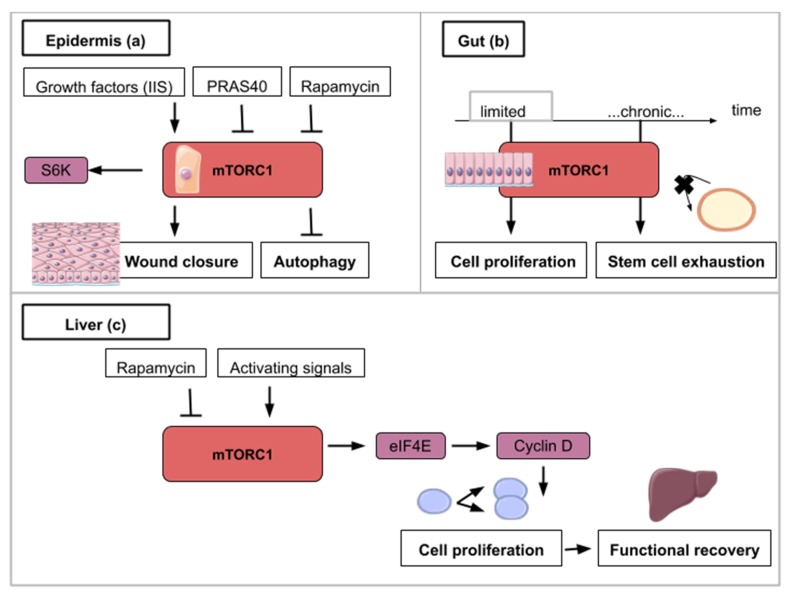
This figure illustrates the ways in which activity from the mTOR signaling pathway contributes to epidermis (**a**), gut (**b**) and liver regeneration (**c**): (**a**) mTORC1 activity is necessary for wound closure and can be enhanced by insulin/insulin-like growth factor signaling (IIS). S6K1/2 is a TOR target which helps wound closure. mTOR inhibitory signals like rapamycin or an overexpressed PRAS40 delay wound closure and promote autophagy. (**b**) For gut regeneration, mTORC1 activity is required for initial cell proliferation but chronic activation leads to intestinal stem cell exhaustion. (**c**) For liver regeneration, mTORC1 activity leads to cell cycle reentry and functional recovery. eIF4E (Eukaryotic Initiation Factor 4E) dependent translation is activated with mTORC1 activity and leads to Cyclin D expression. Cyclin D amplifies cell proliferation and leads to functional liver recovery.

The *Drosophila* monolayered epidermis is also a good system to study tissue repair after injury. One of the first events after injury is the formation of an actomyosin cable that will tighten around the wound. Then, the wound closure is ensured by the association of crawling cells to lamellipodia-carrying cells surrounding the wound. In the next experiments, *Drosophila* larvae were subjected to a laser beam that ablated cells of the epidermis. Wound closure clearly involves Insulin/IGF signaling (IIS) as shown in flies carrying the deletion of three insulin ligands or the expression of a dominant-negative version of the insulin receptor [44,45,46]. Wound healing in *Drosophila* is also delayed in the presence of rapamycin [44] (Figure 4a). Similarly, targeted inhibition of mTORC1 in transgenic mice overexpressing PRAS40 in basal keratinocytes resulted in delayed wound healing [45] (Figure 4a). These animal model studies show that mTOR activity at the site of the wound is required for cell migration and wound healing. The exploitation of the mTOR pathway as a source of novel pharmacological strategies for wound healing is ongoing and is reviewed by Castilho et al. [46].

## 6. The mTOR Signaling Roles during Homeostatic Growth and Stem Cell Maintenance in the Gut Epithelium

Barrier epithelia such as the posterior midgut epithelium of *Drosophila* is another paradigm for the study of tissue repair. One of the steps of *Drosophila*’s midgut regenerative mechanism is the endoreplication of enterocytes (EC). Intestinal stem cells of the gut proliferate to form an enteroblast (EB) population in which cells are able to differentiate into enterocytes (EC) or enteroendocrine cells. As they differentiate, EC undergo endoreplication which contributes to an increase in cell size. The endocycle is different from the mitotic cycle as DNA replication occurs without nuclear division resulting in polyploid cells with increased cell size. Analyzing how endocycling-induced EC growth is used during fly gut epithelium repair following stress, EC endoreplication was shown to be controlled by EGFR/MAPK (epidermal growth factor receptor and mitogen-activated protein kinases respectively) but not by TOR signaling [47]. The EGFR/MAPK pathway promotes cell growth and proliferation. In normal conditions, however, the fly posterior midgut will use the Insulin/PI3K/TOR pathway for EC endocycling and growth [47]. These data serve as an illustration for mTOR being implicated in the physiological homeostatic growth of the gut but not in the regenerative process following epithelium stress. In consequence, the therapeutic activation of mTOR aiming at promoting gut regeneration after mechanical or chemical injury might induce a deleterious effect on the non-affected gut epithelium portion undergoing physiological cell turn-over. This highlights the importance to favor the local administration of drugs over a wide-spread action. This strategy might better target the damaged tissue and preserve the intact epithelium.

The fruit fly intestine hosts intestinal stem cells (ISCs) that proliferate in response to regenerative stimuli. Using enteropathogen infection to mimic regenerative stimuli, fly guts were then dissected and labeled with anti-phospho-4E-BP antibodies. TOR activation was found to be necessary for the quick activation of the proliferation of ISCs [48]. However, the pool of ISCs is significantly reduced when TOR is continuously activated after several rounds of regenerative inputs in the posterior midgut. This decline is explained by an accelerated differentiation of intestinal stem cells into enterocytes and enteroendocrine cells [48]. Similar findings have been observed on stem cells (basal cells) of the mouse tracheal epithelium upon SO_2_ exposure-induced injury: TOR is transiently activated upon chemical-induced damage but chronic activation of TOR induced a decline of basal cells population [48] (Figure 4b). These results outline the dual role of TOR in stem cell homeostasis. It is important to trigger proliferation immediately after damage but a low-grade activity is necessary to ensure long stem cell longevity. This trade-off between mTORC1-mediated inductions of stem cell proliferation and differentiation-induced loss seems to be widely conserved across cell types [26,29,48,49,50,51,52]. Pharmaceutical alterations of the mTOR pathway have to be designed accordingly so as not to deplete adult stem cell populations.

## 7. The mTOR Signaling Role in Hepatocyte Proliferation during Liver Regeneration

Upon partial hepatectomy, the remaining and quiescent mammalian hepatocytes are able to reenter the cell cycle synchronously and proliferate to restore liver volume. The liver does not rely on a resident stem cell population but on the aptitude of its hepatocytes to reactivate proliferation after local signals such as tissue resection. The use of mice mutants (S6K1^−/−^, S6K2^−/−^) and rapamycin intraperitoneal injection to abrogate TOR activity in a 70% partial hepatectomy (PH) mouse model demonstrated the importance of the TOR pathway in the initial proliferation phase after resection [53,54]. More specifically decrease in levels of Cyclin D1, a cyclin known to be involved in the G1-S phase transition, is observed in S6K1^−/−^, S6K2^−/−^ hepatocytes accounting for the cell cycle progression delay reported in these genetically modified hepatocytes. In addition, rescue experiments using Cyclin D1 overexpression in S6K1^−/−^, S6K2^−/−^ hepatocytes showed that cell cycle entry timing can be rectified after PH [53]. The link between mTOR and cell cycle entry can be explained by the fact that mTOR inhibition induces phosphorylation of eIF4E (eukaryotic initiation factor 4E) [55] that participates in the synthesis of the Cyclin D1 protein [56]. In a context of mTOR inhibition, eIF4E-dependant translation is inhibited [55] which results in the decrease of Cyclin D1 protein levels and cell cycle progression delay (Figure 4c). Nevertheless, liver regeneration can happen to a limited extent only. While TOR-mediated proliferation of hepatocytes allows liver regeneration after two-thirds ablation, livers that have suffered 90% hepatectomy are not able to regrow. Unsurprisingly, TOR is not activated in the remnant liver after such massive resection [57].

Liver diseases stem from various etiologies and can be fatal. Therapies enhancing endogenous regeneration following resection should inescapably focus on the activation of the mTOR pathway to create a more favorable microenvironment for hepatocyte reactivation. A recent review cites some of the chemical drugs that stimulate mTOR signaling to improve liver regeneration after partial hepatectomy [5].

## 8. The mTOR Signaling Roles during Bone Formation and Resorption

**Figure 5:**
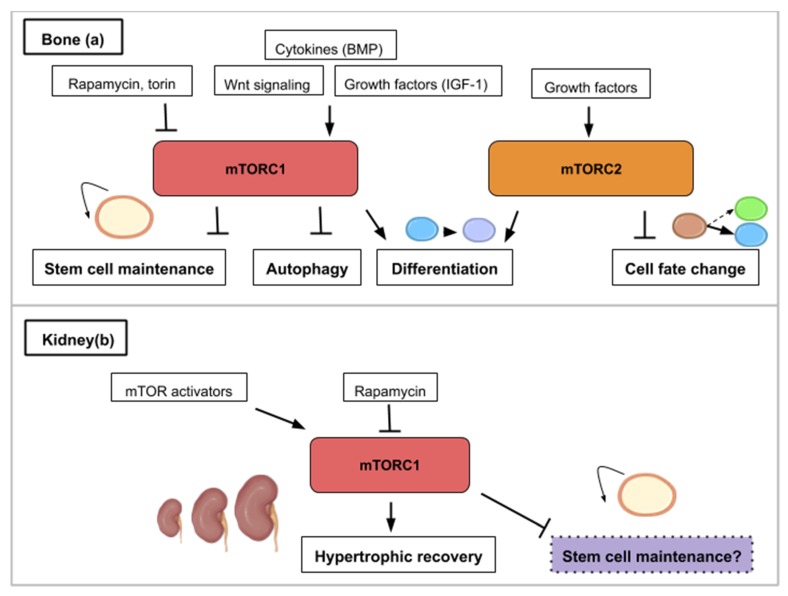
This figure illustrates the ways in which the mTOR signaling pathway contributes to bone (**a**) or kidney (**b**) regeneration. (**a**) mTOR activity (mTORC1 and mTORC2) is required for osteoblast and osteoclast differentiation. mTORC1 is activated by bone morphogenetic protein (BMP), insulin-like growth factor 1 (IGF-1) and Wnt signaling. mTORC1 inhibition promotes stem cell maintenance and autophagy while mTORC2 inhibition leads to cell fate change. (**b**) In the kidney, hypertrophic recovery depends on mTORC1 activity. mTOR activity has been correlated to stem cell depletion such as that mTOR inhibition (purple) might play a role in maintaining kidney progenitors.

Bone regeneration is a physiological process of bone formation and is observed during normal fracture healing and in continuous remodeling and growth throughout an organism’s lifespan. Chen and Long describe the roles of mTOR signaling in skeletal development and disease in a recent review [58]. Osteoblasts are specialized mesenchymal stem cells that differentiate into the major cellular components of bone. Additionally, osteoclasts derive from hematopoietic progenitors from the bone marrow and form the structural components of bone with osteoblasts. Stimulation of osteoblast differentiation has been attributed to mTORC1 as a common effector mediating the bone anabolic effect of insulin-like growth factor-1, Wnt and bone morphogenetic protein (BMP). mTORC1 activity drives bone anabolism by stimulating osteoclast differentiation (Figure 5a). mTORC2 too has been found to promote osteoblast differentiation and function [58] (Figure 5a). In ovariectomized rats, bone marrow stromal cells (BMSC) lacking Rictor gene exhibited reduced osteogenic potential, but an increased capacity to undergo adipogenic differentiation in vitro [59]. Thus, mTORC2 may serve as a potential therapeutic target for treating age-related bone loss.

mTOR modulation has been linked to improve symptoms in certain bone diseases. Bone health is determined by the correct balance of bone resorption and bone formation. Osteoporosis and osteoarthritis are two chronic diseases, which show an imbalance between bone resorption and formation. Osteoarthritis (OA) is a chronic degenerative joint disease characterized by gradual loss of articular cartilage, synovial inflammation, and subchondral bone remodeling. The mTOR pathway has been shown to be overexpressed in human OA chondrocytes [60]. Drugs inhibiting mTORC1 (local application of rapamycin or torin) have shown therapeutic promise [61,62,63,64,65]. One explanation for the pathology is proposed to be linked to the overexpression of mTOR and its induced repression of autophagy [60,64]. The expression of autophagy markers is suppressed in human OA cartilages as well as animals models of OA and the inhibition of autophagy causes chondrocyte apoptosis and OA-like pathogenesis in vitro and in vivo [66,67].

Osteoporosis (OP) is a disease in which the density and quality of bone are reduced. A study in rats showed that rapamycin treatment reduced senile osteoporosis by activating osteocyte autophagy and preserving osteocytes and by a decrease in apoptosis of osteocytes and a decrease in number of osteoclasts [68]. Another study with Everolimus (equivalent to rapamycin) in rats showed a decrease of 60% in cancellous bone loss, the first type of bone structure, which is affected in osteoporosis. Both studies showed that rapamycin or a rapalog decrease osteoporosis by blocking mTOR and decreasing the activity of osteoclasts and the preservation of osteocytes by increasing autophagy. In general, mTORC1 deficiency in osteoclast precursors may promote both osteoclast formation and bone resorption [61].

Bone and dentin derive from stem cells from the apical papilla (SCAP) which are a subpopulation of mesenchymal stem cells (MSCs). SCAP are identified as a population of postnatal mesenchymal stem cells with the capacity for self-renewal and multipotent differentiation into osteoblasts/odontoblasts, adipocytes, and neural cells. Looking specifically at the Phosphoinositide 3 kinase (PI3K)-Akt-mTOR pathway, it was found that its suppression played a role in enhancing the in vivo and in vitro osteogenic/dentinogenic differentiation of stem cells from the apical papilla [69]. A novel approach for SCAP-based bone and dentin regeneration could then involve a suppressive regulation of PI3K-Akt-mTOR signal pathway. This study showed the role of the PI3K-Akt-mTOR pathway in cell fate and stem cell differentiation capacity (Figure 5a). Such data should be taken into account when elaborating possible therapeutic treatments as the mTOR pathway can also play a role in cell fate.

## 9. The mTOR Signaling Roles during Kidney Repair, Disease and Stem Cell Maintenance

The functional unit of the kidney is the nephron and adult neonephrogenesis is unequally distributed in the animal kingdom. The nephron is composed of a glomerulus and a tubule. The glomerulus contains podocytes, which help carry out the initial blood filtration. The tubule is where diverse molecules are secreted and reabsorbed. Adult mammals cannot form new nephrons contrary to cartilaginous fish, bony fish or certain amphibians [70,71,72,73]. However, cellular mechanisms can contribute to the reparation of mammalian renal structures. More specifically, this consists in proliferation of epithelial cells in the tubules—which can originate from dedifferentiated cells (Table 1) or recruited resident progenitor cells—leading to nephron hypertrophy. This repair depends on the ability of the remaining tubular cells to proliferate and restore the injured tubular epithelium [74]. However, the increased intracapillary pressures and flows associated with adaptive glomerular hypertrophy in humans can ultimately lead to podocyte injury and proteinuria [75]. Proper glomerular filtration which regulates blood homeostasis is paramount for functional kidney regeneration. Nephron hypertrophy is mTOR-dependent and rapamycin treatment delays recovery of renal function after acute kidney injury in rats [75]. The effect of rapamycin might be due to the dual effects of inhibition of proliferation and induction of apoptosis of tubular cells.

Medical approaches for treating kidney diseases have been focusing on the stimulation of endogenous reparative mechanisms or on stem cell therapies [76] and the use of organoids [77]. mTOR has emerged as an important modulator of several forms of renal diseases [78]. Activation of mTOR within the kidney occurs in animal models of diabetic nephropathy and other causes of progressive kidney disease. This kinase has also been implicated in the development of glomerular disease, polycystic kidney disease and kidney transplant rejection [78]. Rapamycin ameliorates several key mechanisms believed to mediate changes associated with the progressive loss of glomerular filtration rate in chronic kidney disease [75]. These include glomerular hypertrophy, intrarenal inflammation, and interstitial fibrosis. mTOR also plays an important role in mediating cyst formation and enlargement in autosomal dominant polycystic kidney disease [75]. Inhibition of mTOR by rapamycin or one of its analogues represents a potentially novel treatment for autosomal dominant polycystic kidney disease. mTOR modulators for renal recovery are already ongoing clinical trials and were reviewed in 2018 [79].

During embryogenesis, nephrons are derived from a pool of self-renewing progenitors. Mammalian nephron progenitors stop propagation and are terminally differentiated within a few days after birth [80] indicating that neonephrogenesis is not possible in the adult as mentioned above. In contrast, formation of nephrons *de novo* has been reported in the adult little skate, *Leucoraja erinacea*, an elasmobranch cartilaginous fish, after partial nephrectomy [70]. Similarly, in the adult zebrafish kidney and using transplantation experiments, a small group of cells were discovered as able to form functional nephrons [71,73]. In the skate, histological analysis revealed an enhancement of nephron growth in the nephrogenic zone, both in the remnant tissue and in the contralateral kidney. In addition, stem cell-like mesenchymal cells were identified in the nephrogenic zone [70]. This strongly suggests that a pool of nephron progenitors is maintained in the renal tissue of adult cartilaginous fish giving them the possibility to form new nephrons. What are the molecular properties that allow these renal progenitors to be maintained in the adult? One hypothesis might be that these cells display low rates of protein synthesis. Interestingly, genes/pathways that are differentially expressed in young versus old renal progenitors include pathways with known effects on organism/stem cells aging (such as TOR) and the translational machinery [81] (Figure 5b). More generally, recent reviews have highlighted that both embryonic and adult stem cells are dependent on low rates of translation to maintain an undifferentiated state [4,82]. Pathways controlling protein synthesis such as the mTOR pathway might then have a crucial role in maintaining renal progenitors in adult cartilaginous fish. Retaining a low rate of protein synthesis and more specifically mTOR activity might then be a way to keep renal stem cells in the mammalian adult kidney and to avoid the decline of the progenitor pool around birth. As described in the examples in the bone subsection, mTOR activity effects are characterized by a certain trade-off between stem cell maintenance and differentiation.

## 10. The mTOR Signaling Roles during Whole-Body Regeneration and Asexual Reproduction in Cnidarians, Planarians, and Tunicates

The capability to regenerate the entire body is observed in a wide range of metazoans (mainly platyhelminthes, annelids, echinoderms, tunicates, and cnidarians) but few studies have investigated the role of TOR in this process. This last part of the review will explore mTOR signaling in whole-body regeneration (cnidarian and planarian) and asexual reproduction (tunicate). The freshwater cnidarian *Hydra* is a polyp displaying an oral edge surrounded by tentacles and an aboral, flattened tip permitting substrate attachment. After bisection, this polyp can regrow missing structures through cellular rearrangement and transdifferentiation (see Table 1). With the mTOR complex acting as a nutrient sensor, the mTOR pathway influences cell survival, cell growth, and proliferation as we have illustrated previously. As defined earlier on, autophagy is a catabolic process of cell-component recycling and it plays a fundamental role in cellular, tissular, and organismal homeostasis and has been shown to be involved in regeneration. It is correlated with inhibition of the mTOR pathway or with rapamycin treatment. Like the mTOR pathway components, autophagy proteins are highly conserved in eukaryotic organisms [1].

Initially described as a means of protein degradation [83], autophagy is now understood as holding multiple physiological roles. Beyond the recycling of proteins and macromolecules, mTOR and autophagy in the context of regeneration help determine stem cell maintenance and cell identity [84]. Investigating the role of autophagy during regeneration in *Hydra*, a kinetics of TOR mRNA by semi-quantitative RT-PCR at several time points was performed after amputation [85]. Indeed, it was previously shown that rapamycin induces autophagy in *Hydra* and also in *Drosophila* [86,87]. In the regenerative part of *Hydra*, TOR mRNA levels are maintained stable during the first 4 h post-amputation (hpa) then decrease until 32 hpa which might indicate that autophagy is activated during the first phase of regeneration. TOR mRNA synthesis restarts from 32 to 48 hpa probably reflecting a need for cell growth at this regeneration step [85]. It seems likely that this observed autophagic phase produces the energetic resources from recycled metabolites required to pursue regeneration. In muscle satellite cells, an autophagic flux is also induced in quiescent cells as they activate and enter the cell cycle [88]. In general, stem cell activation is associated with a large increase in cellular ATP, which might then come from an initial autophagic phase. In this example, TOR activity is upregulated only after an autophagic and low mTOR activity phase.

Active mTOR signaling has additionally been shown to be required during tissue regeneration by regulating stem cell activation and blastema outgrowth in the planarian regeneration model [89,90,91]. Planarian flatworms possess an iconic adult stem cell population composed of neoblasts. Following amputation, these neoblasts undergo two rounds of proliferation and migrate to the wound site to form a blastema. Neoblasts then reform missing tissue by differentiation in this blastema which becomes gradually pigmented. In *Schmidtea mediterranea*, TOR and raptor are expressed in neoblasts and their knock-down by RNAi affect the initial mitotic response and blastema formation but not the differentiation that is taking place in existing tissues [89,90,92]. In intact planarians, neoblast proliferation and maintenance are not affected by TOR down-regulation [90] reminding that the effect of TOR on stem cell homeostasis should be interpreted in the context of animal state (normal or regenerating). Finally, a quantitative proteomic analysis has been performed during planarian (*Dugesia japonica*) head regeneration (2 h and 6 h after injury) and uncovers a set of 162 proteins whose expression is modified. During the initial phase of regeneration, these proteins include several molecules related to protein synthesis and mTOR signaling [91] which confirms their key role in priming head regeneration. A certain parallel can be drawn between limb regeneration in mice and axolotl and between whole-body regeneration in planarians. While cells distant from the amputation site are cued to re-enter into the cell cycle (G_alert_), only those residing locally within the stump of the amputated limb ultimately participate in the regrowth of the new limb. During planarian regeneration, neoblasts throughout the body are initially activated, but eventually, this activation becomes refined to the site of injury and blastema growth like in the axolotl or mice models [93]. Whole-body regeneration, as described here with the planarians, informs us that mTOR signaling is a necessary part of the pro-regenerative environment following amputation and that this signaling has a precise temporal dynamic.

**Figure 6:**
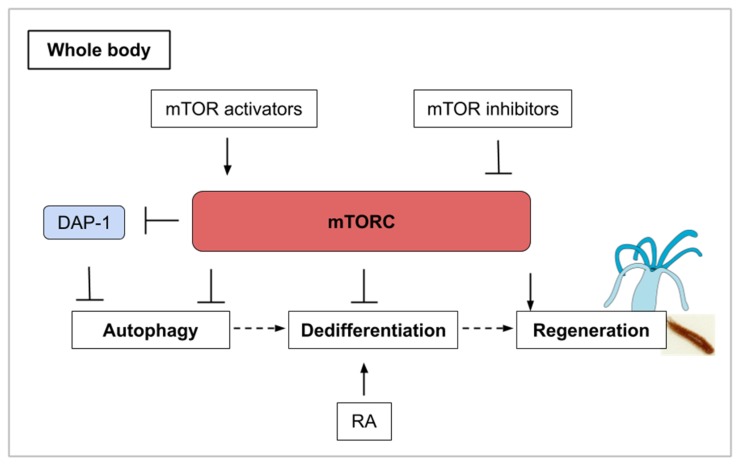
Whole-body regeneration (as explained for cnidarians and planarians) involves different phases of mTORC activity. mTORC inhibition and its induced autophagy are associated with a large increase in cellular ATP which could contribute to stem cell activation. Autophagy is also involved in the process of transdifferentiation, often preceded by dedifferentiation. In combination with mTORC1 inhibition, retinoic acid stimulation induces transdifferentiation. DAP-1 (Death-associated protein 1) inhibits autophagy in conditions of nutrient deprivation. Finally, mTORC activity is upregulated for the regrowth of the missing structure.

Like in *Hydra*, autophagy is activated during the process of regeneration in planarians. Cells that undergo autophagy express Dap-1 (death-associated protein). Loss-of-function experiments of the planarian ortholog of DAP-1 using RNAi were designed to investigate the role of autophagy for regeneration in *Girardia tigrina*. The experiments demonstrated that regeneration is affected in *Dap-1 (RNAi)* animals [92]. Interestingly, Dap-1 is a suppressor of autophagy and also a direct substrate of mTOR [94]. During conditions of nutrient deprivation, mTOR activity is turned off and this causes a reduction in the inhibitory phosphorylation of Dap-1. The mTOR pathway thus contains a brake system in which mTOR inhibition also controls a buffering system that prevents the overactivation of autophagy under nutrient deprivation [94] (Figure 6). This type of self-regulating molecular control mechanism is probably key in deciding cell survival, proliferation and fate for functional regeneration and can represent a challenge in the development of pro-regenerative pharmaceutical strategies.

Budding (also called asexual reproduction, see Table 1) is another way of regeneration that can be observed in cnidarian polyps [95] and urochordates. In the tunicate *P. misakiensis*, this process of regeneration involves the dedifferentiation of cells within the atrial epithelium. Autophagic events occur during dedifferentiation in budding urochordates and could participate in cell remodeling [84]. It has been found that chemical treatment with TOR inhibitors (both PP242 and rapamycin) induced autophagosome formation [96]. The activation of Autophagy-related genes such as *Atg7* was only observed when TOR inhibition was combined with retinoic acid (RA) stimulation. Retinoic acid is a morphogen derived from retinol (vitamin A) that plays important role in cell growth, differentiation, and organogenesis. RA stimulation in tunicates induces transdifferentiation-related genes [96] and secondary bud axis formation [97]. The mTOR and retinoic acid pathways might then act synergistically on bud tissues to coordinate the autophagic events that precede regeneration [96]. Once again, molecular crosstalk is an inevitable issue in drug development and only in vivo experiments for human disease-related treatments can validate new molecules as efficient. In this example of the interplay between autophagy and dedifferentiation, mTOR is illustrated as the orchestrator of various cellular events, which can lead to regeneration in combination with other pathways (Figure 6).

## 11. Conclusions

mTOR signaling has emerged as a pivotal pathway in regulating cell proliferation and differentiation in a wide variety of cell types. Table 2 summarizes the condensed roles of mTOR signaling in different regenerative processes. It is clear that mTOR has an important role in the regulation of tissue homeostasis, in modulating the balance between self-renewal and differentiation, as well as in autophagy. Consequently, it is necessarily involved in regenerative processes. In this review, mTORC1 is more frequently involved in regeneration but a potential bias exists as mTORC1 is better characterized. This is also due to the absence of mTORC2 specific inhibitors. With aberrations in the mTOR axis frequently reported in cancer and various pathologies, a clear understanding of the up-stream and down-stream effectors of mTOR in a tissue-specific or even cell-specific contexts will be crucial in elaborating efficient treatments. Theoretically, many molecules can impact mTOR signaling: Firstly, upstream signals such as cytokines, growth factors or amino-acids; secondly, agents that interact with the mTOR pathway components themselves and finally molecules, which regulate TOR targets. Suitable drugs targeting the mTOR pathway might be designed to work on a specific cell type to avoid side effects. Indeed, an overactivation of mTOR could be responsible for inflammatory reactions such as gliosis in neural parenchyma or inflammatory response associated with chronic kidney diseases. Methods for targeted delivery include nanoparticles with specific coatings such a cell-specific ligands or pH sensitive-molecules [98]. The use of liposomes is another way to target a specific tissue with a drug [99]. On the other hand, continuous activation of mTOR in stem cell populations can lead to premature differentiation and a complete depletion of the stem cell pool while a targeted activation of mTOR in neurons or muscle cells will definitively help these cells to regenerate. The possibility to control when and for how long the drug will be active once administrated should, therefore, be a valuable parameter of its design. Furthermore, understanding the regeneration window, depending on the medical challenge, is also important as treatments need to occur in a regeneration-competent environment. In addition, some regenerative processes seem to involve a dynamic regulation of mTOR activity. This is the case when autophagy is involved in the initial step of tissue recovery. These observations resonate with the concept of the regeneration window described by Dolan et al. [9], where regeneration competency, determined in time and space, is associated with a dynamic molecular orchestra. Finally, the specificities which underlie functional regeneration (conduction, ionic transport, contractility for example) must be taken into account when elaborating treatments for the regrowth of structures lost to human diseases.

From the evolutionary point of view, it is clear that some species are provided with extreme regenerative capacity while others display a more limited aptitude to regrow tissues and structures after amputation or surgical resection. This difference might be explained by the cooption of certain inhibitors of mTOR such as the one hidden in the long non-coding RNA, LINC00961, in damaged mammalian muscles. In contrast, a gradual accumulation of TOR activators by the renal stem cells around birth can explain why the maintenance of this population in the adult mammalian kidney is impaired. The diversity of regeneration processes displayed in the animal kingdom might conclusively constitute a tank for the identification of new TOR modulators that can be next adapted to medical treatments for human diseases.

## Figures and Tables

**Table 1:** Glossary of key terms used in this review.

Definition
**Stem cell differentiation** is the process where a stem cell acquires a specific cell identity.
**Cellular transdifferentiation** refers to the process whereby a differentiated cell changes into another differentiated cell identity.
**Cellular dedifferentiation** is the process where a terminally differentiated cell reverts to a stem cell-like identity.
**Regenerative blastema** is formed after tissue, body or appendage amputation and is composed of a mass of undifferentiated cells displaying the capacity to grow and regenerate the missing part.
**Autophagy** is a lysosome-dependent degradation pathway that allows cells to recycle damaged or superfluous cytoplasmic content such as proteins, organelles, and lipids. Autophagy acts as an intracellular quality control, promotes the viability of cells deprived of nutrients/growth factors and participates in cell remodeling during differentiation.
**Asexual reproduction** in metazoans is a type of reproduction by which a new individual arises directly from a single organism. It does not involve the fusion of gametes.

**Table 2:** Summary of the implication of the mTOR pathway in different regenerative processes.

Regenerative Process	Role of mTOR in Regeneration	Animal Model and References
Appendage	mTORC1 is required and mTOR genes are differentially expressed during regeneration.	Teleost [16,17]
Muscle	mTORC1 controls an alert satellite cell state, is required for cell proliferation and differentiation and for myofiber growth.	Mouse [18,20,21,23,24,25,26], Pig [22], Axolotl [27]
Axon	mTORC1 activity stimulates axon survival, growth and functional recovery after injury of the central nervous system. mTOR inhibition serves regeneration by inhibiting gliosis.	Mouse [31,32,33,35,36,41,43], Chick [30], Human [28,29] Rat [33], *Drosophila* [38,39],
Epidermis	mTORC1 activity activates cell proliferation and wound repair.	*Drosophila* [44,45]
Gut	mTOR activity is necessary for gut cell growth and proliferation but continued activation leads to stem cell exhaustion.	*Drosophila* [47,48], Mouse [48]
Liver	mTOR activity is required for hepatocyte proliferation and leads to functional recovery for up to 70% partial hepactectomy.	Mouse [53,54,57], Zebrafish [100]
Bone	mTORC1 activity mediates osteoblast and osteoclast differentiation and stem cell maintenance. mTORC2 activity influences cell fate.	Mouse [58,59,61], Human [69]
Kidney	mTORC1 mediates hypertrophic response following kidney injuries.	Rat [75,79]
Whole-body	mTOR genes are downregulated during pre-regenerative autophagy but activity is required for initial mitotic response and blastema formation.	*Hydra* [85,86], Planarian [89,90,91,92], Urochordate [65]

## Abbreviations

mTOR	mechanistic/mammalian Target of Rapamycin
FGF	Fibroblast Growth Factor
JAK-STAT	Janus kinase—Signal Transducers and Activators of Transcription
TGF-β	Transforming Growth Factor-beta
TORC1	Target of Rapamycin Complex 1
TORC2	Target of Rapamycin Complex 2
Raptor	Regulatory-associated protein of mammalian target of rapamycin
Rictor	Rapamycin-insensitive companion of mTOR
S6K1/2	S6 kinase 1/2
4E-BP	4E binding protein
Sin1	Stress-activated map kinase-interacting protein 1
IGF/IGF-1	Insulin-like growth factor/Insulin-like growth factor—1
SHH	Suppression subtraction hybridization
iPSC	Induced pluripotent stem cells
SPAR	Small regulatory polypeptide of amino acid response
HeLa	Henrietta Lacks cells
PC3	Prostate Cancer cells
v-ATPase	Vacuolar-type H^+^-ATPase
CNS	Central nervous system
RGC	Retinal ganglion cells
PTEN	Phosphatase and tensin homolog
GSK3ß	Glycogen synthase kinase 3
RTP801	Responsive protein (or Redd1)
MGPC	Müller glia-derived progenitor cells
NMDA	N-methyl-D-aspartate
OPN	Osteopontin
AAV	Adeno-associated virus
MB	Mushroom body
TSC-Rheb-Tor	Tuberous sclerosis complex- Ras homolog enriched in brain—Target of rapamycin
UNF	Unfulfilled Nuclear Receptor
PI3K	Phosphoinositide 3-kinase
SCI	Spinal Cord Injuries
ISS	Insulin/IGF signaling
PRAS40	Proline-Rich Akt Substrate of 40 kDa
FOXO	Forkhead Box O (transcription factors)
IL-2	Interleukine 2
ISC	Intestinal Stem Cells
SO_2_	Sulfur dioxide
EB	Enteroblast
EC	Enterocyte
EGFR/MAPK	Epidermal Growth Factor Receptor/Mitogen-Activating Protein Kinases
PH	Partial Hepatectomy
eIF4E	Eukaryotic Initiation Factor 4E
E2	17β-estradiol
GPER1	G Protein-Coupled Estrogen Receptor 1
BMP	Bone Morphogenetic Protein
BMSC	Bone Marrow Stromal Cells
SCAP	Stem Cell from Apical Papilla
PKD	Polycystic Kidney Disease
ATG	Autophagy related gene
RT-PCR	Reverse Transcriptase—Polymerase Chain Reaction
hpa	Hours post amputation
Dap-1	Death Associated Protein
RA	Retinoic Acid
